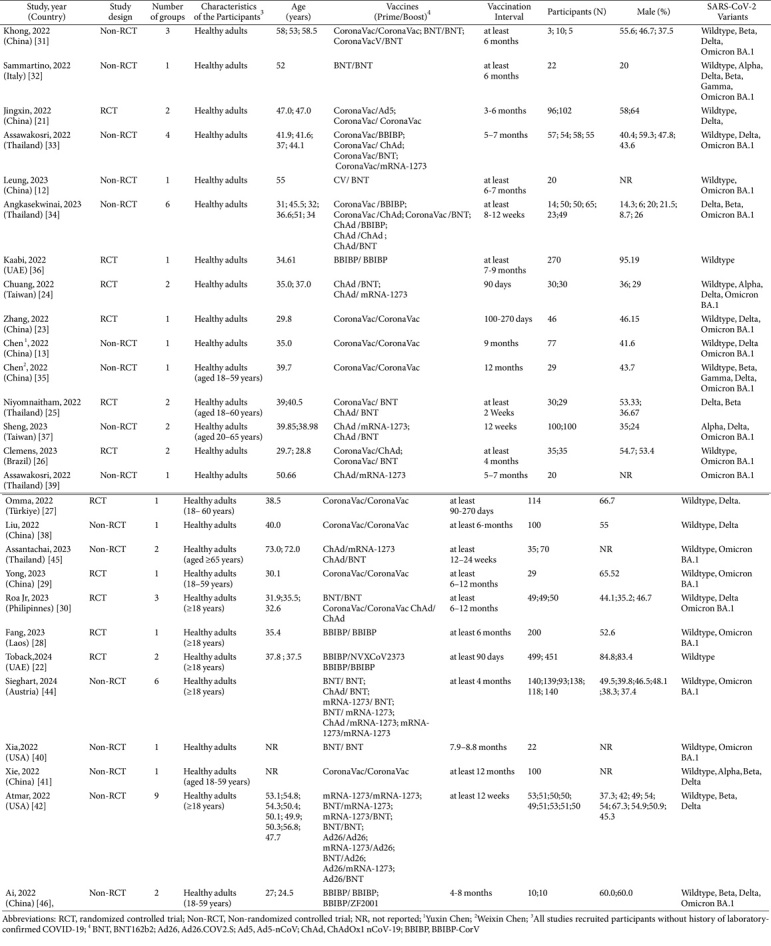# A Meta-Analysis on the Immunogenicity of Homologous versus Heterologous Immunization Regimens against SARS-CoV-2 Beta, Delta, and Omicron BA.1 VoCs in Healthy Adults

**DOI:** 10.4014/jmb.2411.11059

**Published:** 2025-02-24

**Authors:** Jo-Lewis Banga Ndzouboukou, Abdul A. Kamara, Nadeem Ullah, Qing Lei, Xiong-lin Fan

**Affiliations:** 1 Department of Pathogen Biology, School of Basic Medicine, Tongji Medical College and State Key Laboratory for Diagnosis and Treatment of Severe Zoonotic Infectious Diseases, Hubei Key Laboratory of Drug Target Research and Pharmacodynamic Evaluation, Huazhong University of Science and Technology, Wuhan, P.R. China; 2 Department of Mathematica and Statistics, Fourah Bay College, University of Sierra Leone, Sierra Leone; 3 Department of Clinical Microbiology, Umeå University 90187, Umeå, Sweden; 4 Division of Nephrology, Tongji Hospital, Tongji Medical College, Huazhong University of Science and Technology, Wuhan, P.R. China

**Keywords:** COVID-19 vaccines, SARS-CoV-2 VoCs, homologous booster, heterologous booster, immunogenicity, neutralization titers

## Abstract

Since the outbreak of the COVID-19 pandemic, SARS-CoV-2 has not stopped evolving, leading to the emergence of variants of concern (VoCs) involved in significant immune escape. Here, we compared the immunogenicity of different prime-boost vaccination regimens against SARS-CoV-2 wildtype (WT) and its Beta, Delta, and Omicron BA.1 VoCs. We used 5 databases to retrieve publications and random-effect models to estimate pooled neutralization titers. We included 11 randomized controlled trials (RCTs) and 16 non-RCTs, 10 prime-boost vaccination regimens, and 4598 subjects. We found neutralization activity against SARS-CoV-2 decreased with virus evolution. The heterologous immunization was more effective. The increase in neutralization titers against SARS-CoV-2 WT and Beta, Delta, and Omicron BA.1 VoCs after heterologous immunization was 1.41(95%CI:0.82–2.01), 0.90(95%CI:0.39–1.41), 1.23 (95%CI: 0.81–1.65), and 1.32 (95%CI: 0.99–1.65), respectively. Furthermore, the booster dose of viral vector vaccine did not show a higher increase in neutralization titers against SARS-CoV-2 WT(MD=0.48; 95%CI:−1.12-1.09), Beta (MD=0.20; 95%CI:−0.26-0.67), Delta (MD=0.35; 95%CI:−0.09-0.79), and Omicron BA.1 (MD=0.38; 95%CI:−0.14-0.89) VoCs. The combination of inactivated-recombinant protein vaccines showed a higher increase in neutralization titers (Beta: MD=1.88 and Delta: MD=1.70) than other combinations of vaccines. However, only a combination of mRNA-viral vector vaccines showed a higher increase in neutralization titers (MD:1.52; 95%CI:0.34-2.70) against Omicron BA.1 VoC. Interestingly, the viral vector-mRNA immunization regimen appears better compared to mRNA-viral vector regimen, especially against Beta and Delta VoCs. Overall, the type of combination followed by the order of administration of COVID-19 vaccines could be a potential vaccine strategy against the occurrence of SARS-CoV-2 variants.

## Introduction

More than 4 years ago, clusters of patients with signs of pneumonia (*e.g.*, fatigue or lassitude, cough, fever, chest pain, etc.) of unknown origin were signalized in Wuhan City (Hubei province, China). The etiological agent was rapidly identified and classified as a new type of Coronavirus (CoV), then later named severe acute respiratory syndrome coronavirus 2 (SARS-CoV-2), and recognized as the causal agent of COVID-19 [1]. SARS-CoV-2 is evolutionarily related to other potentially deadly respiratory viruses, including the SARS-CoV-1 and Middle East respiratory coronavirus virus (MERS-CoV), both from the beta-CoV genus and *Coronaviridae* family [2]. Referring to the World Health Organization (WHO) statistics, cumulative confirmed COVID-19 cases and deaths have reached 776,9 million and 7,07 million, respectively [3]. The rapid transmission of COVID-19 has sparked deep research activity to recognize possible treatments, including examination of the current drugs and the development of vaccines [3]. Several COVID-19 vaccines, including recombinant protein subunit, inactivated virus, viral vectored, and messenger ribonucleic acid (mRNA) vaccine platforms, have been approved by WHO for emergency use against SARS-CoV-2 infection, each featuring varying degrees of efficacy and acceptable safety [4]. To date, only 12 vaccines have been authorized by the WHO for using in fight against COVID-19 [5]. Like all RNA viruses, SARS-CoV-2 undergoes many changes (mutations) during its evolution [6]. Over the last COVID-19 pandemic, many SARS-CoV-2 variants have appeared, and have been classified by WHO as variants of concern (VoCs), including Alpha (B.1.1.7), Beta (B.1.351), Gamma (P.1), Delta (B. 1.617.2) and Omicron BA.1 (B.1.1.529)[7]. Furthermore, several studies have indicated that VoCs have a high transmission rate, and COVID-19 vaccines have shown reduced effectiveness against VoCs [8]. These VoCs may compromise the efficacy of COVID-19 vaccines, following numerous mutations present in their spike (S) protein [9]. All of these resulted in an enhancing effect on the binding of the Receptor-Binding Domain (RBD) of VoCs to human Angiotensin-Converting Enzyme 2 (hACE2) receptors compared to SARS-CoV-2 WT, suggesting that structural epistasis allows for significant immune escape while maintaining efficient receptor engagement [10]. To address the major challenges posed by VoCs, vaccination with a booster of the COVID-19 vaccine has been suggested and evaluated in clinical trials and observational studies [11]. Although various studies on COVID-19 booster immunizations have been conducted to evaluate the cumulative effectiveness of COVID-19 vaccines [12, 13], however, the results of the efficacy of homologous and heterologous booster immunization against VoCs need to be updated mostly when more studies on COVID-19 vaccines to prevent VoCs infection become available. Therefore, our study was designed to compare the immunogenicity of heterologous and homologous prime-booster immunization regimens against SARS-CoV-2 WT and its Beta, Delta, and Omicron BA.1 VoCs to know which is the best combination of heterologous vaccines against SARS-CoV-2 WT, and its VoCs; and the best order of administration of COVID-19 vaccines to establish a better strategy in administering COVID-19 vaccine boosters.

## Materials and Methods

### Data Sources and Search Strategy

This study was conducted following PRISMA 2020 guidelines [14]. A meticulous search strategy was performed in 5 international electronic medical databases, including PubMed, medRxiv, Scopus, Google Scholar, and Cochrane Library, to identify relevant studies regarding the immunogenicity of prototype COVID-19 vaccines in healthy adults. We performed the search of eligible studies using the Population, Intervention, Comparison, and Outcome (PICO) approach to determine suitable keywords, establish search terms, and define study eligibility criteria [15]. The use of a combination of the following search terms was considered to form the search strategies: ‘‘COVID-19 vaccines”, ‘‘SARS-CoV-2 variants”, ‘’Immunization’’,‘’ Vaccination’’, ‘’Efficacy’’, ‘‘Homologous’’, ‘‘Heterologous”, ‘‘Booster”, ‘’Neutralizing antibodies’’, ‘’SARS-CoV-2 variants” from January 2022 to August 31, 2024.

### Study Selection

Studies investigating neutralizing antibodies (NAbs) against SARS-CoV-2 Beta, Delta, and Omicron BA.1 VoCs were included, nonetheless of language or publication status. Using plaque reduction neutralization assay (PRNT), followed by surrogate virus neutralization test (sVNT), and then pseudotyped virus neutralization test (pVNT) [16], all included studies in the meta-analysis assessed the neutralization of each VoCs by serum antibodies and compared neutralization titers against each VoCs with those of the SARS-CoV-2 WT. Serum samples were obtained from immunized subjects who received a booster immunization program, nevertheless, the type of vaccine received. The inclusion criteria for the screening of relevant studies were as follows:

1. Studies reporting immunogenicity in healthy adults without a history of COVID-19 infection or comorbidities, and who received 2 doses of homologous vaccines.

2. Studies evaluating immunogenicity or COVID-19 vaccine efficacy.

3. Studies that included subjects who received the first booster dose of COVID-19 vaccines.

4. RCTs and non-RCTs published in English language.

Priority was given to serum neutralization titers before (baseline; day 0) and after 14 days post-vaccination. However, when data at 14 days after booster immunization were unavailable, data at 28 days post-vaccination were selected as the endpoint. Neutralization activity against SARS-CoV-2 VoCs following a booster dose was measured by comparing the change of neutralization titers. Only the most recent and more detailed studies were included in the event of overlapping data.

We excluded all studies that meet the exclusion criteria described as follows:

1. Studies conducted on the subjects aged <18 years.

2. Other categories of studies, like animal studies, protocol, conference abstracts, reviews, systematic reviews, commentaries or meta-analyses.

3. Studies with poor information regarding the method or unit of immunogenicity assessment.

4. Studies that do not specify the type of COVID-19 vaccines administered to the subjects, and original articles published in another language other than English.

5. We excluded studies presenting data in graphical format because of the difficulty related to the assessment of immunogenicity outcomes.

### Data Collection Process

Search results from each database were grouped to remove duplicate articles. Following a pre-developed extraction form in Microsoft Excel 365, two authors (J.BN and N.U) independently extracted data in duplicate, to evaluate the eligibility of studies referring to established inclusion and exclusion criteria:

1. Study characteristics (*e.g.*, Name of the first author, year of study publication, country...).

2. Participant characteristics (*e.g.*, mean/median age, prime-boost immunization regimen…)

3. Interventions (*e.g.*, COVID-19 vaccine booster dose…),

4. Comparisons (*e.g.*, type of COVID-19 vaccines, before and after the booster vaccination…),

5. Outcomes, including immunogenicity profile (*e.g.*, level of NAbs titers against SARS-CoV-2 WT, Beta, Delta, and Omicron BA.1…).

When the eligible articles did not supply the required information in the way we expected, we calculated them by hand according to the available data. In addition, we have tried to polish the data collection by reading supplementary materials to avoid missing information. Disagreements among authors were immediately resolved following a comprehensive discussion among authors or through a decision of another author (XF).

### Endpoints

The primary outcome was the evaluation of the long-term immunogenicity or protection following post-prime vaccination. The secondary outcome was the levels of NAb activity against SARS-CoV-2 WT and its Beta, Delta, and Omicron BA.1 VoCs post-booster vaccination. The third outcome evaluated the best combination of heterologous COVID-19 against SARS-CoV-2 WT and its VoCs. The fourth outcome evaluated the best order of prime-boost COVID-19 vaccine administration. Neutralization titers were evaluated by PRNT, pVNT, or sVNT for SARS-CoV-2 WT and its VoCs at baseline and 14/28 days after receiving the third dose. For NAbs, we tore out the standard deviation (SD) and geometric mean titers (GMT). We transformed to ‘’Mean’’ and ‘’SD’’ all data from studies which published the NAbs level as median or range utilizing the method suggested by the Cochrane Handbook [17]. In this meta-analysis, full vaccination or immunization is determined as immunization following a prime vaccine series. A booster is determined as a supplemental dose after full immunization.

### Risk of Bias and Quality Assessment

Two reviewers (J.BN and NU) separately evaluated the risk of bias about each included study, which was cross-checked by QL and XF. For RCT, a modified tool for evaluating risk-of-bias for randomized trials (RoB-2) tool was used, as endorsed by the Cochrane Collaboration [18]. The biases were examined into 5 main domains: (i) the randomization process, (ii) deviations from intended interventions, (iii) missing outcome data, (iv) measurement of the outcome, and (v) selection of the reported result. The studies were classified as low risk, with some concerns, or high risk of bias. For non-RCTs, a Newcastle–Ottawa Scale (NOS) risk of bias tool was used [19]. The NOS comport 3 main groups (8 subgroups). A score of 0–3, 4–6, and 7–10 stars was regarded as low, moderate, or good quality study, respectively.

### Statistical Analysis Procedure

This meta-analysis compares the neutralization activity of SARS-CoV-2 WT against its VoCs, in prime and booster vaccination regimens. The GMT of NAbs was extracted from eligible studies, and the geometric mean difference (MD) was used to compare the difference in immunogenicity. Neutralization titers were log-transformed (*Log*_10_) prior to the analysis. Forest plots were used to indicate the point estimates of the MD and 95%confidence interval (95%CI) compared among heterologous and homologous primary series vaccinations. We evaluated heterogeneity among studies utilizing the χ^2^ -based Q test and I^2^ statistical parameter. The I^2^ test indicates heterogeneity as low (I^2^ ≤ 40%), moderate (I^2^ > 40% to < 60%), and high (I^2^ > 60%) [20]. Statistically significant results were considered at *p* < 0.05. We utilized the random-effects inverse variance method which incorporates an estimate of the between-study variance to compute the weights for the model. Moreover, all pooled results were stratified between groups of the immunization regimens. We utilized Z-tests to compare the differences in immunogenicity among different immunization regimens. All statistical analyses were executed by utilizing the meta package (version 4.0.5) of R under the environment of R-Studio (version 1.4.1103). All data provided and analyzed in this study were from electronic medical databases; consequently, institutional review board approval was not required.

## Results

**Fig. 1:**
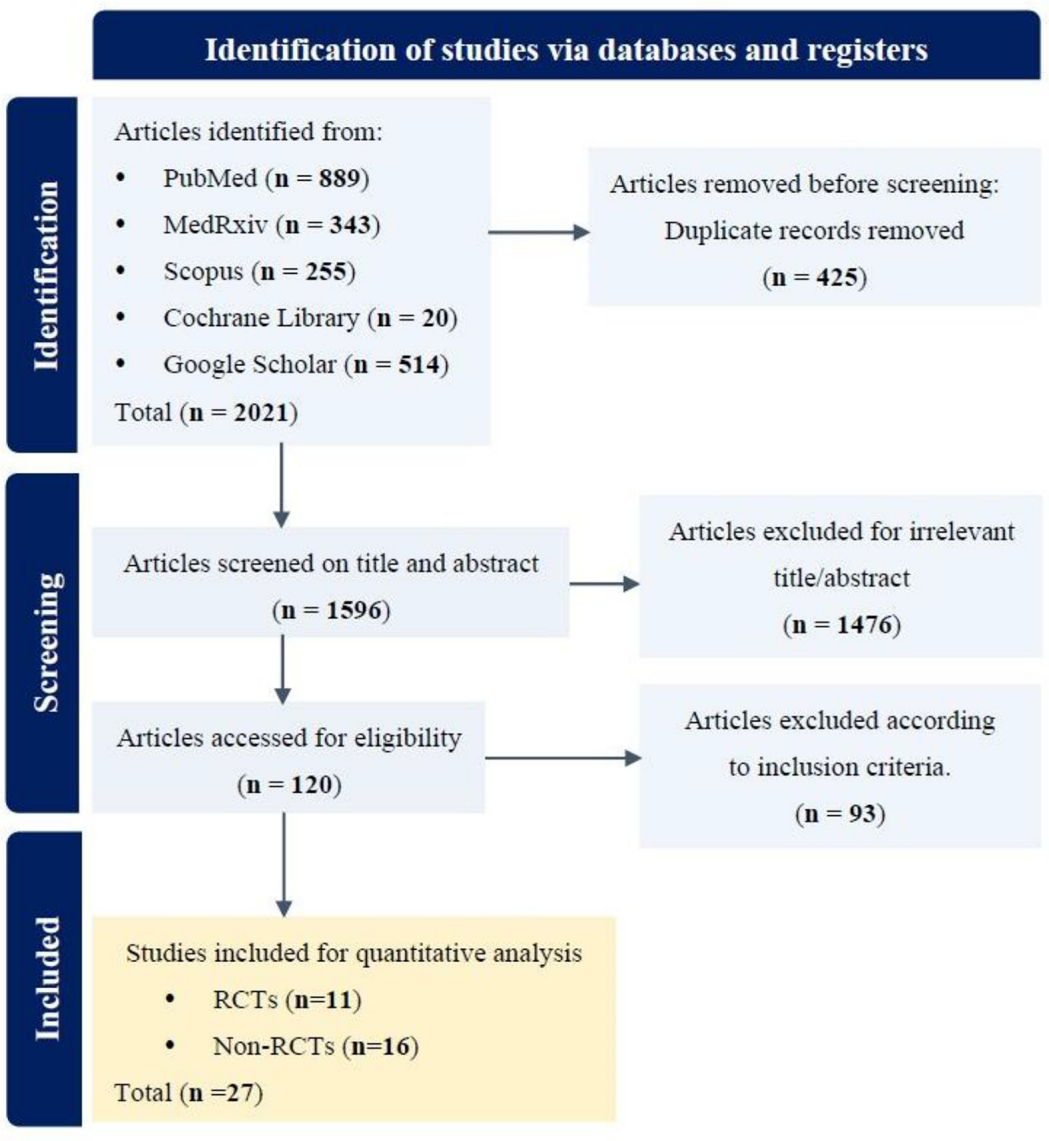
Flowchart for article searches and selection strategies.

A total of 2,021 studies were recovered following an initial search of Cochrane Library (n =20), Scopus (*n* = 255), medRxiv (*n* = 343), Google Scholar (*n* = 514), and PubMed (*n* = 889). We removed 425 studies because of duplications. Based on a review of the titles/abstracts, two reviewers filtered 1476 studies and incorporated the remaining 120 for the entire text review. After a full-text review, 27 studies [12, 13, 21-45] were finally incorporated into this meta-analysis according to the eligibility criteria for study inclusion (Fig. 1).

### Characteristics of Identified Studies

Every eligible study utilized in this meta-analysis was published between January 2022, and August 31, 2024. There were various study designs for included studies, 11 RCTs [21-30, 36], and 16 non-RCTs (cohort studies) [12, 13, 31-35, 37-45]. Ten studies were conducted in China [12, 13, 23, 29, 31, 35, 38, 41, 43, 46], five studies in Thailand [25, 33, 34, 39, 45], two studies in the USA [40, 42], two studies in the UAE [28, 36], two studies in Taiwan [24, 37], one study in Turkey [27], one study in Brazil [26], one study in Austria [44], one study in Laos [28], one study in Italia [32], and one study in Philippines [30]. In total, 10 COVID-19 vaccines (CoronaVac, BBIBP-CorV, NVX-CoV2373, Soberana Plus, ZF2001, Ad5-nCoV, Ad26.COV2.S, ChAdOx1-nCoV-19, mRNA-1273, and BNT162b2) and 3 SARS-CoV-2 VoCs described in our previous study were involved [4]. CoronaVac (Sinovac) and BBIBP-CorV (Sinopharm) are inactivated vaccines; ZF2001 (Zhifei Longcom), NVX-CoV2373 (Novavax), and Soberana Plus (BioCubaFarma) are Recombinant protein vaccines; ChAdOx1-nCoV-19 (AstraZeneca), Ad5-nCoV (CanSino), and Ad26.COV2.S (Janssen) are viral vector vaccines; BNT162b2 (Pfizer-BioNTech) and mRNA-1273 (Moderna) are mRNA vaccines [4].

We described 8 specific groups including homologous (Inactivated-Inactivated, mRNA-mRNA, viral vector-viral vector) or heterologous (inactivated-mRNA, inactivated-viral vector, inactivated-recombinant protein, mRNA–viral vector, and viral vector–mRNA) prime-boost regimens.

The baseline information on included studies for analysis are listed in Table 1, and a total of 4598 participants representing the study population were adults without a history of COVID-19 infection. Thirteen of 16 non-RCTs studies were judged as good quality, and the remaining 3 were of moderate quality (Table S1). The status of all included studies, assessed through NOS, has also been provided (Table S1). All the RCTs were judged as low risk for overall risk-of-bias judgment (Table S2).

### Evaluation of Long-Term Neutralization Activity against VoCs Post-Prime Vaccination

**Fig. 2:**
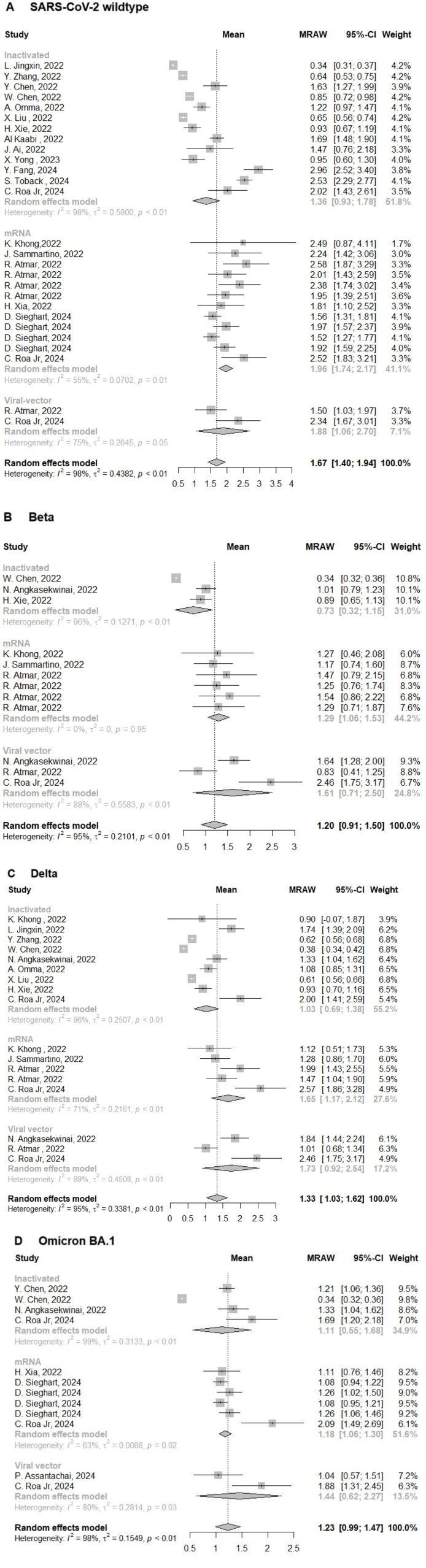
Forest plot showing the pooled log-transformed neutralization titers against: (**A**) SARS-CoV-2 WT, and its (**B**) Beta, (**C**)Delta, and (**D**) Omicron BA.1 VoCs before booster vaccination. Weight is an indicator of the impact of each study on the overall results. MRAW, mean raw; 95%-CI, 95% confidence interval; I^2^, index for the degree of heterogeneity between studies. τ^2^, measure of heterogeneity. Squares represent effect sizes for each study, and rhombus represent pooled results for all studies.

As expected, few weeks following the prime COVID-19 vaccination, our results revealed that the levels of NAbs titers against SARS-CoV-2 WT, and its Beta, Delta, and Omicron BA.1 VoCs were 1.67(95% CI: 1.40-1.94, I^2^ =98%; *p* < 0.01), 1.20(95% CI: 0.91–1.50, I^2^ = 95%; *p* < 0.01), 1.33(95% CI: 1.03–1.62, I^2^ = 95%; *p* < 0.01), and 1.23(95% CI: 0.99-1.47, I^2^ = 98%; *p* < 0.01), respectively (Fig. 2A-2D). In the general population, neutralization activity against SARS-CoV-2 WT and its VoCs significantly declined following the SARS-CoV-2 evolution. Besides, we observed a significant reduction in neutralization activity against VoCs, particularly in individuals immunized with both inactivated vaccines (Fig. 2A-2D). In addition, we divided the COVID-19 vaccines used in this study into 3 groups following the vaccine platform to evaluate the variation in their long-term immunogenicity. As expected, we found that the individuals primed with both mRNA (mRNA-1273 and BNT162B2) vaccines exhibited the highest level of NAbs titers against SARS-CoV-2 WT (MRAW_WT_ = 1.96, 95% CI: 1.74–2.17, I^2^ =55%)(Fig. 2A). However, about SARS-CoV-2 Beta, Delta, and Omicron BA.1 VoCs, we found that the long-term immunogenicity of the viral vector vaccines (MRAW_Beta_ = 1.61, 95% CI:0.71-2.50, I^2^ = 88%; *p* < 0.01; MRAW_Delta_ = 1.73, 95% CI: 0.92-2.54, I^2^ = 89%; *p* < 0.01; MRAW_Omicron BA.1_ = 1.44, 95% CI: 0.62-2.27, I^2^ = 80%; *p* < 0.01) was much better than mRNA, and inactivated vaccines included in this study (Fig. 2B-2D).

### Evaluation of Neutralization Activity against VoCs Following Homologous Post-Boosters Vaccination

**Fig. 3:**
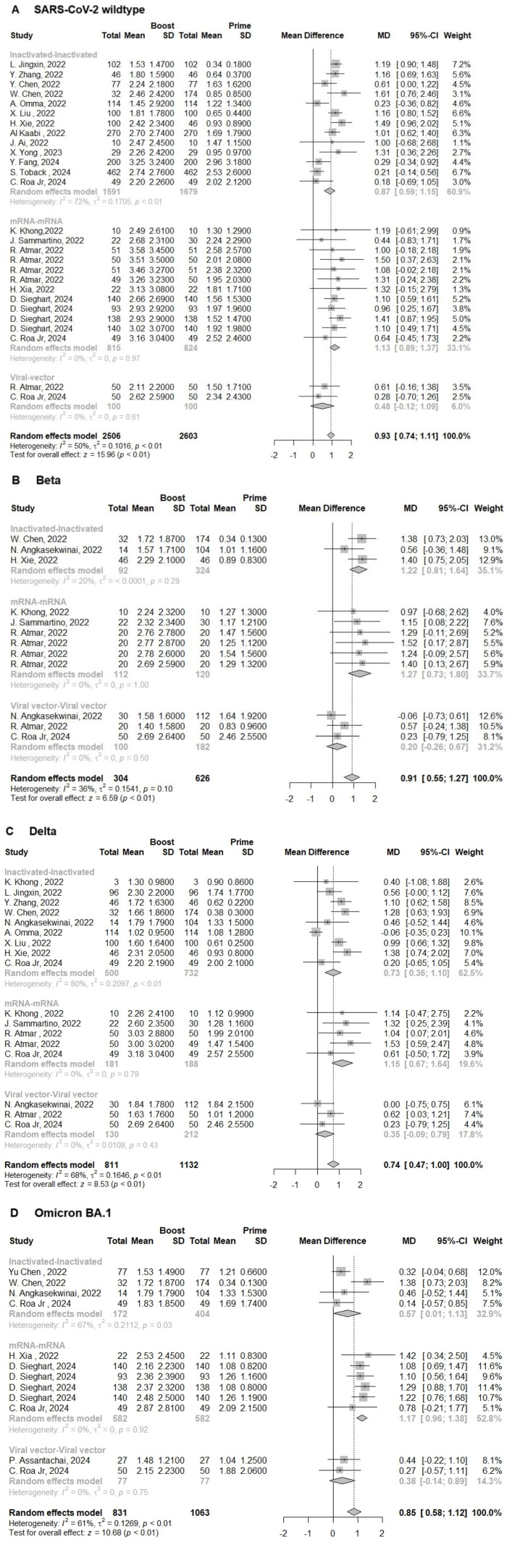
Forest plot showing the pooled log-transformed neutralization titers against: (**A**)SARS-CoV-2 WT, and its (**B**) Beta, (**C**)Delta, and (**D**) Omicron BA.1 VoCs before(Prime) and after homologous booster vaccination (Boost). Weight is an indicator of the impact of each study on the overall results. 95%-CI, 95% confidence interval; I^2^, index for the degree of heterogeneity between studies; τ^2^, measure of heterogeneity; SD, standard deviation; MD, mean difference; Squares represent effect sizes for each study, and rhombus represents pooled results for all studies.

To evaluate neutralisation activity following homologous immunization regimens, we compared neutralisation titers against SARS-CoV-2 WT and its VoCs before and then after a booster (third) dose. The improvement of neutralization titers against SARS-CoV-2 WT and its Beta, Delta, and Omicron BA.1 VoCs was 0.93(95% CI: 0.74-1.11, I^2^ = 50%), 0.91(95% CI: 0.55-1.27, I^2^ = 36%), 0.74(95% CI: 0.47-1.00, I^2^ = 68%), and 0.85(95% CI: 0.58-1.12, I^2^ = 61%) at 14- or 28-days following administration a third dose, respectively (Fig. 3A-3D). However, with the virus evolution, only the individuals who received homologous mRNA vaccines as boosters had a remarkable increase in neutralization titers. The neutralization titers against SARS-CoV-2 WT, and its Beta, Delta, and Omicron BA.1 VoCs were 1.13(95% CI: 0.89-1.37, I^2^ = 0%), 1.27(95% CI: 0.73-1.80, I^2^ = 0%), 1.15(95% CI: 0.68-1.63, I^2^ = 0%), and 1.17(95%CI: 0.96-1.38, I^2^ = 0%), respectively (Fig. 3A-3D). Concerning the individuals who received homologous inactivated and viral vector vaccines as booster doses, we observed that a booster dose could not keep them safe from the Omicron BA.1 VoC (MD = 0.57, 95% CI: 0.08-0.76, I^2^ = 54%) and (MD = 0.38, 95%CI: −0.14-0.89, I^2^ = 0%), respectively (Fig. 3D). Interestingly, we found that the people who received third dose of viral vector vaccine as a booster did not show a higher improvement of neutralization titers against the SARS-CoV-2 WT (MD = 0.48, 95% CI: −1.12-1.09, I^2^ = 0%; *p* < 0.01), and its Beta (MD = 0.20, 95% CI: −0.26-0.67, I^2^ =0 %; *p* < 0.01), Delta (MD = 0.35, 95% CI: −0.09-0.79, I^2^ = 0%; *p* < 0.01), and Omicron BA.1 (MD = 0.38, 95%CI:-0.14-0.89, I^2^ = 0%; *p* < 0.01) VoCs (Fig. 3A-3D). In general, regardless of VoCs, the final neutralization titers of homologous mRNA prime-boost immunization were significantly more elevated compared to inactivated and viral vector vaccines (*p* < 0.05). Among the individuals immunized with 2 doses of mRNA vaccines, the levels of NAb titers against SARS-CoV-2 WT, and its VoCs Beta, Delta, and Omicron BA.1 at 14 or 28 days post-booster immunization raised to 2.99 (95% CI: 2.78-3.19, I^2^=0%), 2.59 (95% CI:2.11-3.06, I^2^=37%), 2.92(95% CI: 2.51-3.34, I^2^=0%) and 2.37(95% CI: 2.18-2.56, I^2^=65%), respectively (Fig. S1A-S1D).

### Evaluation of Neutralization Activity against VoCs Following Heterologous Post-Boosters Vaccination

**Fig. 4:**
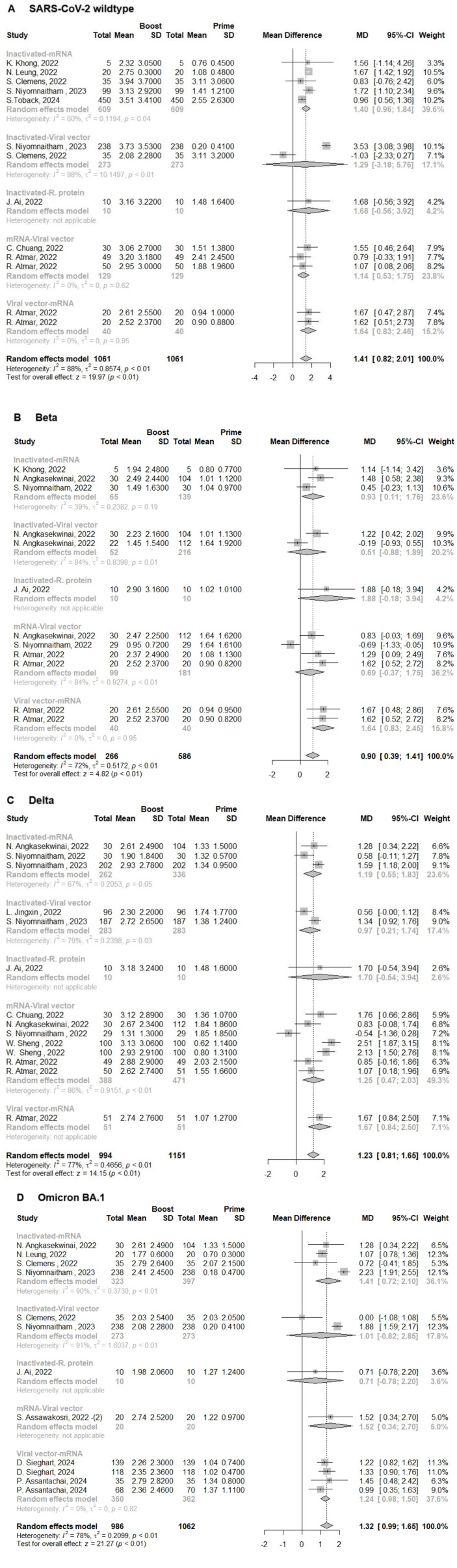
Forest plot showing the pooled log-transformed neutralization titers against: (**A**) SARS-CoV-2 WT, and its (**B**) Beta, (**C**)Delta, and (**D**) Omicron BA.1 VoCs before (Prime) and after heterologous booster vaccination (Boost). Weight is an indicator of the impact of each study on the overall results. 95%-CI, 95% confidence interval; I^2^, index for the degree of heterogeneity between studies; τ^2^, measure of heterogeneity; SD, standard deviation; MD, mean difference; Squares represent effect sizes for each study, and rhombus represents pooled results for all studies.

This section of the study was based on 5 groups of heterologous immunization regimens (inactivated–mRNA, viral vector–mRNA, inactivated–viral vector, mRNA–viral vector and inactivated–recombinant protein). According to the types of immunization regimens, we found that all individuals developed neutralization activity against SARS-CoV-2 WT and its VoCs Beta, Delta, and Omicron BA.1 following immunization with a heterologous booster dose. The pooled improvement of neutralization titers against SARS-CoV-2 WT, and its Beta, Delta, and Omicron BA.1 VoCs was 1.41(95% CI: 0.82-2.01, I^2^ = 88%), 0.90(95% CI: 0.39-1.41, I^2^ = 72%), 1.23(95% CI: 0.81-1.65, I^2^ = 77%) and 1.32 (95%CI:0.99-1.65, I^2^ = 78%), respectively (Fig. 4A-4D). Following 14 or 28 days of a heterologous booster dose, the neutralization titers of SARS-CoV-2 WT, and its Beta, Delta, and Omicron BA.1 VoCs were 2.96(95% CI: 2.63-3.30, I^2^ = 77%), 2.02(95% CI: 1.61-2.44, I^2^ = 76%), 2.59(95% CI: 2.28-2.89, I^2^ = 70%) and 2.23(95%CI: 2.03-2.43, I^2^ = 64%), respectively (Fig. S2A-S2D). Consequently, every heterologous immunization regimen was significantly higher than the homologous regimens (*p* < 0.01). Additionally, we compared the variation in neutralization activity among each heterologous immunization regimen to determine which combinations of heterologous vaccination were most effective against VoCs following homologous prime vaccination. Interestingly, we found that the individuals who received recombinant protein vaccines as booster doses following prime immunization with homologous inactivated vaccines had a higher increment in neutralization titers whether those primed with other types of vaccine combinations for Beta (MD = 1.88; 95% CI: -0.18-3.94) (Fig. 4B) and Delta (MD = 1.70; 95% CI: -0.54-3.94) VoCs (Fig. 4C). However, for the Omicron BA.1 VoC, the individuals who received viral vector vaccines as booster doses following prime immunization with homologous mRNA vaccines had the higher increment in neutralization titers (MD = 1.52; 95% CI: 0.34-2.70) whether those primed with other types of vaccine combinations (Fig. 4D).

Finally, we assess the order of vaccine administration. Our results revealed that the individuals who received the viral vector vaccine regimens as the prime vaccination followed by mRNA vaccine regimens (viral vector-mRNA) as booster dose had the potent highest increments in neutralization titers compared to those who received mRNA vaccine regimens as the prime vaccination followed by viral vector regimen (mRNA-Viral vector) as booster dose, especially against SARS-CoV-2 Beta (MD = 1.64; 95% CI: 0.83-2.45) and Delta (MD = 1.67; 95% CI: 0.84-2.50) VoCs (Fig. 4B and 4C). However, regarding the SARS-CoV-2 Omicron BA.1 VoCs, we found opposite results (MD = 1.52; 95% CI: 0.34-2.70) to those previously mentioned (Fig. 4D). Besides, we could not ignore that even for individuals who received heterologous vaccination regimens, the amelioration in neutralization activity also indicated a higher decrease with the virus evolution.

## Discussion

The appearance of new SARS-CoV-2 variants constitutes major challenges for researchers in the achievement of efficient vaccines to fight COVID-19 [47]. In this meta-analysis implying 27 studies and 4598 subjects, our results showed that neutralization activity against SARS-CoV-2 declined seriously as the virus evolved. Also, we notably observed an important decline in the neutralization activity in populations immunized with inactivated vaccines against SARS-CoV-2 Beta, Delta, and Omicron BA.1 VoCs following the prime immunization. These findings were similar to a precedent study conducted by Duan *et al*. [48]. Indeed, they demonstrated that the decline observed in COVID-19 vaccine effectiveness was due to selective pressure (an important stage in the process of the appearance of viral mutations), thus promoting mutations that give SARS-CoV-2 to evade immune responses. Several studies have shown that the S protein is a crucial target site for NAbs [49], and they are mainly used to determine the efficacy of vaccines [50]. Consequently, the accumulation of S protein mutations results in its immune evasion from neutralization after immunization [8, 49]. The SARS-CoV-2 Beta, Delta, and Omicron BA.1 VoCs contains several variations located in the RBD of the S protein that have more consequential impacts on the neutralizing ability of vaccine-elicited antibodies [43]. For example, the SARS-CoV-2 Omicron BA.1 VoC contains 15 mutations in its RBD and escapes a large number of NAbs subsets [51], which is related to a rise in the transmission rate [52].

Numerous studies have analyzed homologous and heterologous prime-boosting immunogenicity with different COVID-19 vaccine platforms. All of them concluded that the heterologous prime-boosting strategy with COVID-19 vaccines was well tolerated and elicited stronger immune responses than homologous vaccination [53-55]. Besides, Cheng *et al*. [56] followed by Lu *et al*. [57] observed low NAb responses against SARS-CoV-2 Omicron BA.1 VoC in participants who previously received 2 or 3 doses of CoronaVac. In addition, it has been shown that mRNA-BNT162b2 booster dose improves NAbs against SARS-CoV-2 Omicron BA.1 VoC when compared to a primary series of CoronaVac [56, 58], and to a high level than participants immunized with a primary series of mRNA-BNT162b2 [59]. Our findings were similar to these previous observations, as we found that a booster dose with homologous (only mRNA vaccines were associated with a consistent amelioration of the neutralization activity following the virus evolution) or heterologous immunization induces neutralization titers against SARS-CoV-2 Beta, Delta, and Omicron BA.1 VoCs with heterologous immunization showing higher immunogenicity.

As demonstrated by Zuo and colleagues [60], our results also showed that heterologous prime-boost strategy using mRNA vaccine like a booster dose in the participants previously immunized with 2 doses of inactivated vaccines may produce a potent immune response to combat SARS-CoV-2 WT, and its Beta and Delta VoCs. Our findings suggest that using the mRNA vaccine as the booster dose in people who were previously immunized with 2 of the Inactivated vaccines may elicit stronger protection against severe COVID-19 when compared to 3 doses of the inactivated vaccine. The feasible explication of these results could be that mRNA vaccines induce further focused CD4^+^ and CD8^+^ T-cell stimulation against the S protein. In contrast, inactivated vaccines induce a more diffuse response, targeting a large number of various proteins, as mRNA vaccines presented the S proteins as the unique antigen while CoronaVac presented the entire virus [61]. Our data suggested that one shot booster after 2 doses of CoronaVac is enough to boost the memory B-cell response [62]. Boosting along with heterologous vaccines induces the memory B cells to evolve through affinity maturation, leading to a large and strong NAbs response against SARS-CoV-2 VoCs [58]. In a pandemic situation, it is necessary to take unusual measures, such as combining various vaccine platforms with different immunological mechanisms to upgrade protection. Furthermore, our findings agree with another study conducted by Cerqueira-Silvain *et al*. [63], in a Brazilian cohort. Indeed, they showed that administration of the mRNA-BNT162b2 vaccine following CoronaVac vaccine enhanced protection against SARS-CoV-2 infection than the CoronaVac vaccine. The CoronaVac two-dose schedule showed an efficacy of 34.7% against new infections than unvaccinated participants, and this protection decreased over time. However, using mRNA-BNT162b2 vaccine as a booster dose improved protection against infection through 82.6% [63]. Nevertheless, our analysis also showed that not all heterologous boost vaccines could substantially raise NAbs against the SARS-CoV-2 Omicron BA.1 VoC. Our results may have direct implications for developing countries presently using inactivated vaccines as the principal vaccination strategy.

Additionally, we found that only viral vector vaccines may provoke long-lasting immune responses compared to the other COVID-19 vaccines. Interestingly, all individuals immunized with a booster dose of viral vector vaccine did not point to a higher improvement in neutralization titers against the SARS-CoV-2 VoCs. A conceivable explication could be the immune response to the adenovirus vector backbone (anti-vector immunity)[64]. Particularly, various studies observed that pre-existing anti-adenovirus immunity of the participants could influence the vaccine’s safety and immunogenicity [64]. Besides, another study demonstrated that all individuals showing elevated baseline NAbs to Ad5-nCoV were more tolerant to receive a booster dose [64].

As demonstrated by Ai and colleagues [46], we found that the individuals who received recombinant protein vaccines as booster doses following prime immunization with inactivated vaccines had a higher increment in neutralization titers than those primed with other vaccine combinations for SARS-CoV-2 Beta and Delta VoCs. These results suggest that the “priming” of 2 doses inactivated vaccines may induce long-lasting humoral and cellular immunity, which a third booster dose could successfully recall protecting against SARS-CoV-2 WT and its VoCs. Ai and colleagues[46] showed that after two doses of inactivated vaccines as the “priming” shot, using a heterologous booster dose of protein subunit vaccine was sure and strongly immunogenic for participants with no history of COVID-19, which remarkably remind and improved immune responses against SARS-CoV-2 WT and its VoCs. Our results supply significant pieces of evidence for elaborate a future global heterologous boosting scheme against COVID-19. In addition, Liao and colleagues [65] showed that a third dose of the ZF2001 vaccine safely supplied a considerable rise in immune responses by 3- to 16-fold when administered 3–9 months following the second dose of CoronaVac in adults aged 18–59 and ≥60 years [65]. Our results suggest that the interval time among prime and boost can affect the effectiveness of COVID-19 vaccines. These results showed that 2 doses of inactivated vaccine induced lasting humoral immunity that was successfully remind by a booster dose of the recombinant protein. Overall, our results suggest that the utilization of heterologous prime-boost vaccination with various platforms of COVID-19 vaccines is a good strategy, particularly in locations where the stock of vaccines is limited or where vaccine distribution is difficult.

Finally, concerning each type of booster vaccine, our results revealed that the prime-boost order seemed to matter. Indeed, we found that the immunogenicity of the viral vector-mRNA immunisation regimen was improved compared to the mRNA-viral vector regimen, especially against SARS-CoV-2 Beta and Delta VoCs. However, regarding the SARS-CoV-2 Omicron BA.1 VoC, we found opposite results. These findings were in line with the studies conducted by Chiu and colleagues [55] followed by Nordström and colleagues[66]. Indeed, both studies demonstrated that the heterologous mRNA(BNT162b2)-primed + ChAdOx1 nCoV-19-boosted combination was less potent and less immunogenic compared to the homologous mRNA(BNT162b2)-primed and boosted combination, while the ChAdOx1 nCoV-19-primed + mRNA(BNT162b2)-boosted combination elicited stronger immunogenicity against SARS-CoV-2 Alpha, Beta and Delta VoCs [55, 66]. Moreover, they found heterologous ChAdOx1 nCoV-19-primed + mRNA(BNT162b2)-boosted combination induced a stronger T-cell response and highest NAbs between all combinations [55, 66]. These findings demonstrate the complexity of the immune response, and it is not possible to predict the homologous versus heterologous response without clinical trial comparisons. Our findings could be used to improve vaccination strategies against SARS-CoV-2. Therefore, additional studies are needed to discover the suitable order of immunizations.

This study had some limitations. Firstly, for all included studies in the meta-analysis, neutralisation titers were evaluated following dissimilar methods. However, Sholukh *et al*. [67], and Sun *et al*. [68] showed that the elevated agreement among the results of PRNT and pVNT sustained valid cross-study comparisons employing these platforms. Second, the interval time among prime-boost affects the immunogenicity of the COVID-19 vaccines [55]. This is probably the reason which could explain the elevated heterogeneity observed in this study. Thirdly, as the studies were gathered following the type of COVID-19 vaccines, however, some groups contained more studies than others. Fourthly, in some studies the sample sizes were too small.

## Conclusion

This study summarised the immunogenicity of ancestral COVID-19 vaccines against the SARS-CoV-2 wildtype and its VoCs in various prime-boost immunization regimens. Subsequent to the SARS-CoV-2 evolution, neutralization activity against SARS-CoV-2 VoCs has declined, with populations immunized with inactivated COVID-19 vaccines having shown an alarming decline in the neutralization activity. Although a booster dose has shown a significant improvement in the neutralization titers, however, homologous vaccination regimens have progressively been losing their efficacy. The heterologous boost approach can improve protection against SARS-CoV-2 WT, and its VoCs. In addition, even though we found that the immune response against Omicron BA.1 VoC after a booster dose was not enough potent as that against other VoCs, nevertheless some individuals could profit from it. Finally, the combination and the order of COVID-19 vaccine platforms administration seems to play an essential role in the fight against COVID-19. Overall, our results could be an additional potential vaccine strategy against the appearance of SARS-CoV-2 variants or other CoVs. However, assessment of more studies are needed to endorse and identify the optimal combination and the order of administration of different COVID-19 vaccines.

## Supplemental Materials

Supplementary data for this paper are available on-line only at http://jmb.or.kr.



## Figures and Tables

**Table 1:** Characteristics of the original studies included in the meta-analysis.