# Supplementary material for: A Meta-Analysis on the Immunogenicity of Homologous versus Heterologous Immunization Regimens against SARS-CoV-2 Beta, Delta, and Omicron BA.1 VoCs in Healthy Adults
Source: J Microbiol Biotechnol. 2025 Feb 24;35:e2411059. doi: 10.4014/jmb.2411.11059 (PMC11985416; doi:10.4014/jmb.2411.11059)
Supplement: Supplementary file 1 [file jmb-35-e2411059-supple.pdf]

J. Microbiol. Biotechnol. 2025. 35: e2411059
https://doi.org/10.4014/jmb.2411.11059

Supplementary Tables and Figures

Table S1. Risk of bias for included non-RCTs.

	Selection				Comparability	Exposure				
	Item 1	Item 2	Item 3	Item 4	Item 5	Item 6	Item 7	Item 8	Total score	**Quality**
K. Khong, (2022)	0	1	0	1	1	1	1	1	6	Moderate
J. Sammartino (2022)	1	1	1	0	0	1	1	1	6	Moderate
S. Assawakosri (2022)	1	1	1	1	2	1	1	1	9	Good
N. Leung (2023)	1	1	1	1	2	1	1	1	9	Good
N. Angkasekwinai (2023)	1	1	1	0	2	1	1	1	8	Good
Y. Chen (2022)	1	1	1	1	1	1	1	1	8	Good
W. Chen (2022)	1	1	1	0	2	1	1	1	8	Good
W. Sheng (2023)	1	1	1	0	2	1	1	1	8	Good
S. Assawakosri (2022)	1	1	0	1	0	0	1	1	5	Moderate
X. Liu (2023)	1	1	1	0	1	1	1	1	7	Good
P. Assantachai (2023)	1	1	1	0	1	1	1	1	7	Good
D. Sieghart (2024)	1	1	1	0	1	1	1	1	7	Good
H. Xia (2022)	1	1	1	0	2	1	1	1	8	Good
H. Xie (2022)	1	1	1	1	1	1	1	1	8	Good
R. Atmar (2022)	1	1	1	0	2	1	1	1	8	Good
J. Ai (2022)	1	1	1	1	2	1	1	1	9	Good

Item 1: Representativeness of the exposed cohort.
Item 2: Selection of the non-exposed cohort.
Item 3: Ascertainment of exposure.
Item 4: Demonstration that outcome of interest was not present at the start of the study.

Item 5: Comparability of cohorts based on the design or analysis.
Item 6: Assessment of outcome.
Item 7: Was follow-up long enough for outcomes to occur.
Item 8: Adequacy of follow-up of cohorts.

Table S2. Risk of bias of the included studies, assessed using the Cochrane RoB-2 tool.

Study (First author)	Randomization process	Deviations from intended interventions	Missing outcome data	Measurement of the outcome	Selection of the reported result	Overall risk of bias
L. Jingxin (2022)	Low risk	Low risk	Low risk	Low risk	Low risk	Low risk
C. Chuang (2022)	Low risk	Low risk	Low risk	Low risk	Low risk	Low risk
Y. Zhang (2022)	Low risk	Low risk	Low risk	Low risk	Low risk	Low risk
S. Niyomnaitham (2022)	Low risk	Low risk	Low risk	Low risk	Low risk	Low risk
S. Clemens (2023)	Low risk	Low risk	Low risk	Low risk	Low risk	Low risk
X. Yong (2023)	Low risk	Low risk	Low risk	Low risk	Low risk	Low risk
Al. Kaabi (2022)	Low risk	Low risk	Low risk	Low risk	Low risk	Low risk
A. Omma (2022)	Low risk	Low risk	Low risk	Low risk	Low risk	Low risk
C. Roa Jr (2024)	Low risk	Low risk	Low risk	Low risk	Low risk	Low risk
Y. Fang (2023)	Low risk	Low risk	Low risk	Low risk	Low risk	Low risk
S. Toback (2024)	Low risk	Low risk	Low risk	Low risk	Low risk	Low risk

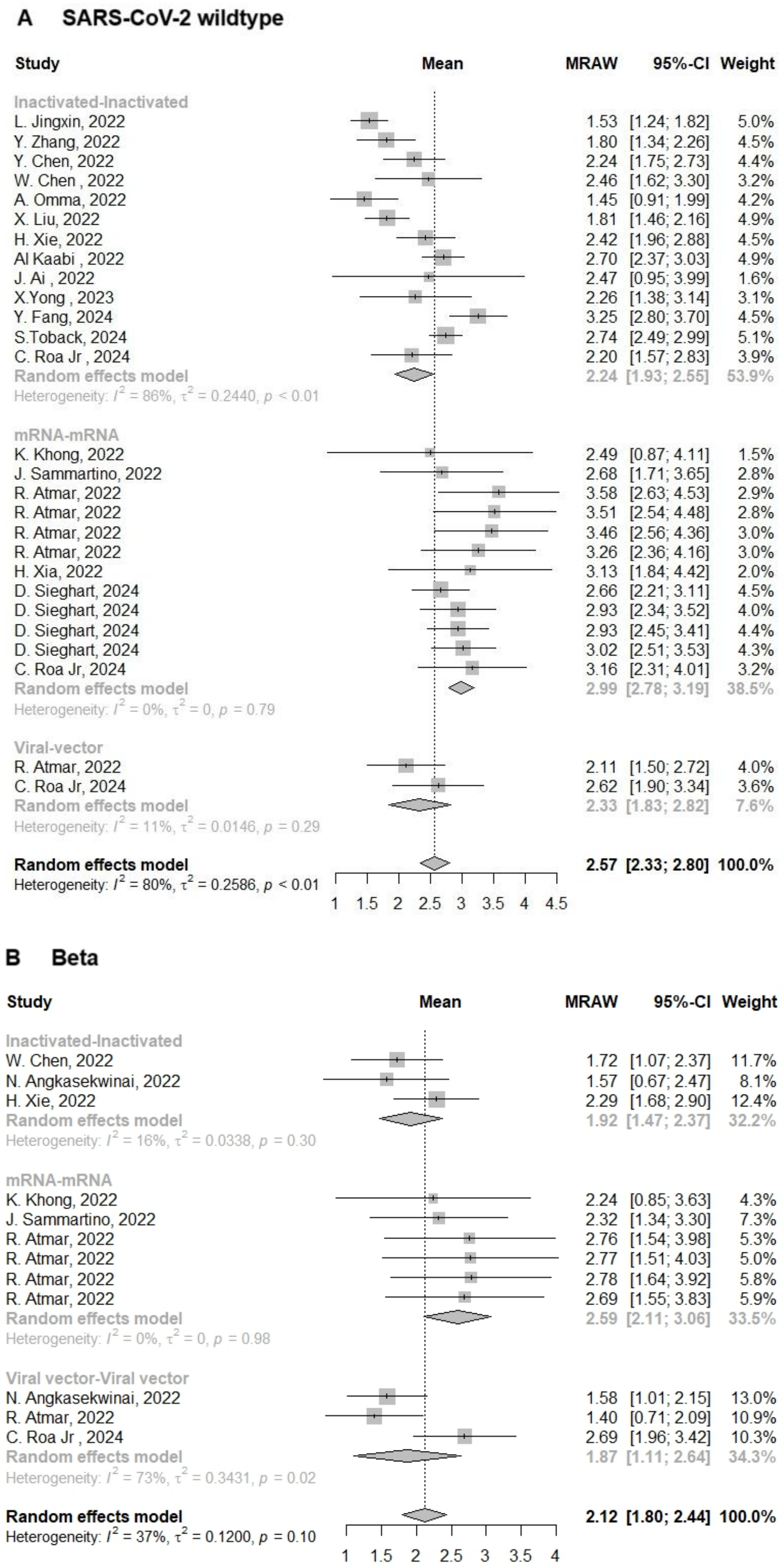
A SARS-CoV-2 wildtype
Study
Mean
MRAW
95%-CI
Weight
Inactivated-Inactivated
L. Jingxin, 2022 1.53 [1.24; 1.82] 5.0%
Y. Zhang, 2022 1.80 [1.34; 2.26] 4.5%
Y. Chen, 2022 2.24 [1.75; 2.73] 4.4%
W. Chen , 2022 2.46 [1.62; 3.30] 3.2%
A. Omma, 2022 1.45 [0.91; 1.99] 4.2%
X. Liu, 2022 1.81 [1.46; 2.16] 4.9%
H. Xie, 2022 2.42 [1.96; 2.88] 4.5%
Al Kaabi , 2022 2.70 [2.37; 3.03] 4.9%
J. Ai , 2022 2.47 [0.95; 3.99] 1.6%
X.Yong , 2023 2.26 [1.38; 3.14] 3.1%
Y. Fang, 2024 3.25 [2.80; 3.70] 4.5%
S.Toback, 2024 2.74 [2.49; 2.99] 5.1%
C. Roa Jr , 2024 2.20 [1.57; 2.83] 3.9%
Random effects model 2.24 [1.93; 2.55] 53.9%
Heterogeneity: I^2 = 86%, τ^2 = 0.2440, p < 0.01
mRNA-mRNA
K. Khong, 2022 2.49 [0.87; 4.11] 1.5%
J. Sammartino, 2022 2.68 [1.71; 3.65] 2.8%
R. Atmar, 2022 3.58 [2.63; 4.53] 2.9%
R. Atmar, 2022 3.51 [2.54; 4.48] 2.8%
R. Atmar, 2022 3.46 [2.56; 4.36] 3.0%
R. Atmar, 2022 3.26 [2.36; 4.16] 3.0%
H. Xia, 2022 3.13 [1.84; 4.42] 2.0%
D. Sieghart, 2024 2.66 [2.21; 3.11] 4.5%
D. Sieghart, 2024 2.93 [2.34; 3.52] 4.0%
D. Sieghart, 2024 2.93 [2.45; 3.41] 4.4%
D. Sieghart, 2024 3.02 [2.51; 3.53] 4.3%
C. Roa Jr, 2024 3.16 [2.31; 4.01] 3.2%
Random effects model 2.99 [2.78; 3.19] 38.5%
Heterogeneity: I^2 = 0%, τ^2 = 0, p = 0.79
Viral-vector
R. Atmar, 2022 2.11 [1.50; 2.72] 4.0%
C. Roa Jr, 2024 2.62 [1.90; 3.34] 3.6%
Random effects model 2.33 [1.83; 2.82] 7.6%
Heterogeneity: I^2 = 11%, τ^2 = 0.0146, p = 0.29
Random effects model 2.57 [2.33; 2.80] 100.0%
Heterogeneity: I^2 = 80%, τ^2 = 0.2586, p < 0.01
1 1.5 2 2.5 3 3.5 4 4.5
B Beta
Study
Mean
MRAW
95%-CI
Weight
Inactivated-Inactivated
W. Chen, 2022 1.72 [1.07; 2.37] 11.7%
N. Angkasekwinai, 2022 1.57 [0.67; 2.47] 8.1%
H. Xie, 2022 2.29 [1.68; 2.90] 12.4%
Random effects model 1.92 [1.47; 2.37] 32.2%
Heterogeneity: I^2 = 16%, τ^2 = 0.0338, p = 0.30
mRNA-mRNA
K. Khong, 2022 2.24 [0.85; 3.63] 4.3%
J. Sammartino, 2022 2.32 [1.34; 3.30] 7.3%
R. Atmar, 2022 2.76 [1.54; 3.98] 5.3%
R. Atmar, 2022 2.77 [1.51; 4.03] 5.0%
R. Atmar, 2022 2.78 [1.64; 3.92] 5.8%
R. Atmar, 2022 2.69 [1.55; 3.83] 5.9%
Random effects model 2.59 [2.11; 3.06] 33.5%
Heterogeneity: I^2 = 0%, τ^2 = 0, p = 0.98
Viral vector-Viral vector
N. Angkasekwinai, 2022 1.58 [1.01; 2.15] 13.0%
R. Atmar, 2022 1.40 [0.71; 2.09] 10.9%
C. Roa Jr , 2024 2.69 [1.96; 3.42] 10.3%
Random effects model 1.87 [1.11; 2.64] 34.3%
Heterogeneity: I^2 = 73%, τ^2 = 0.3431, p = 0.02
Random effects model 2.12 [1.80; 2.44] 100.0%
Heterogeneity: I^2 = 37%, τ^2 = 0.1200, p = 0.10
1 1.5 2 2.5 3 3.5 4

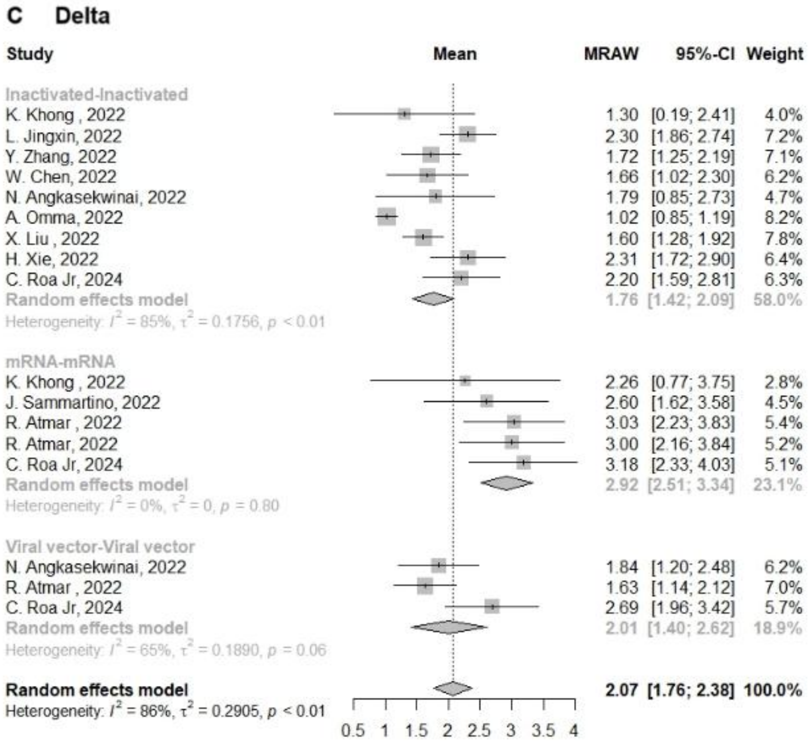

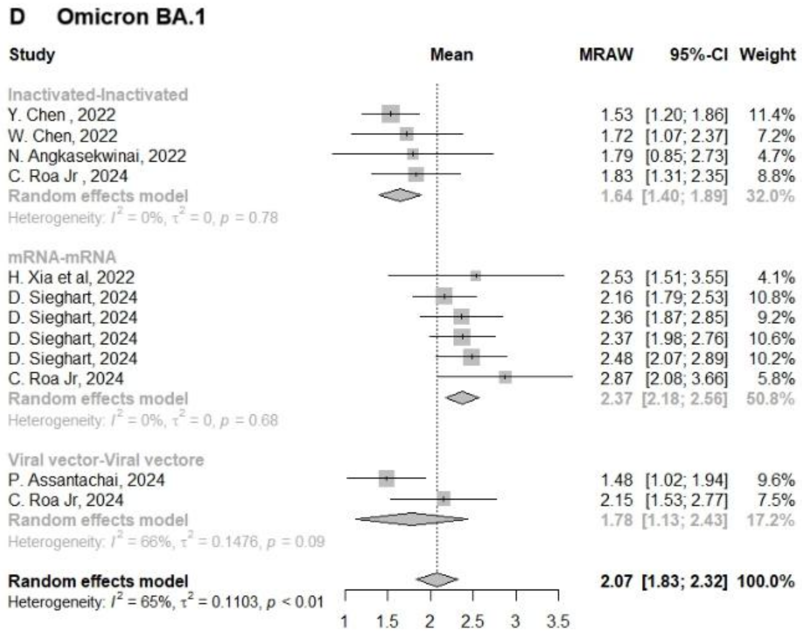

Fig. S1. Forest plot of the pooled log-transformed neutralization titers against (A) SARS-CoV-2 wildtype, (B) Beta, (C)Delta, and (D) Omicron BA.1 after homologous booster vaccination. MRAW, mean raw; 95%-CI, 95% confidence interval; I^2, index for the degree of heterogeneity between studies. τ^2, measure of heterogeneity. Weight is an indicator of the impact of each study on the overall results. Squares represent effect sizes for a single study, and rhombus represent pooled results for all studies.

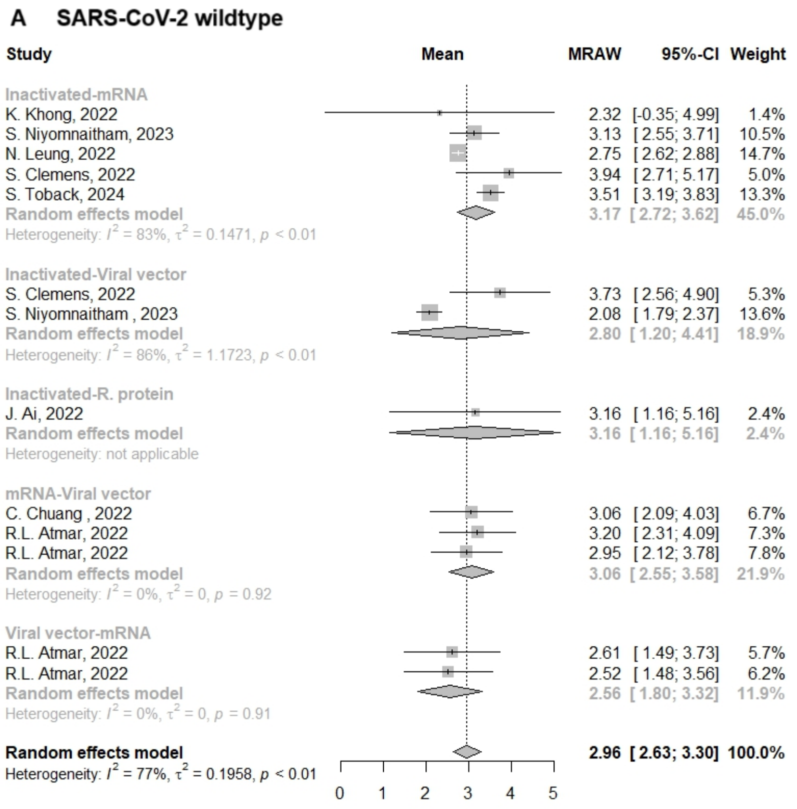
A SARS-CoV-2 wildtype
Study
Mean
MRAW
95%-CI
Weight
Inactivated-mRNA
K. Khong, 2022 2.32 [-0.35; 4.99] 1.4%
S. Niyomnaitham, 2023 3.13 [2.55; 3.71] 10.5%
N. Leung, 2022 2.75 [2.62; 2.88] 14.7%
S. Clemens, 2022 3.94 [2.71; 5.17] 5.0%
S. Toback, 2024 3.51 [3.19; 3.83] 13.3%
Random effects model 3.17 [2.72; 3.62] 45.0%
Heterogeneity: I^2 = 83%, τ^2 = 0.1471, p < 0.01
Inactivated-Viral vector
S. Clemens, 2022 3.73 [2.56; 4.90] 5.3%
S. Niyomnaitham , 2023 2.08 [1.79; 2.37] 13.6%
Random effects model 2.80 [1.20; 4.41] 18.9%
Heterogeneity: I^2 = 86%, τ^2 = 1.1723, p < 0.01
Inactivated-R. protein
J. Ai, 2022 3.16 [1.16; 5.16] 2.4%
Random effects model 3.16 [1.16; 5.16] 2.4%
Heterogeneity: not applicable
mRNA-Viral vector
C. Chuang , 2022 3.06 [2.09; 4.03] 6.7%
R.L. Atmar, 2022 3.20 [2.31; 4.09] 7.3%
R.L. Atmar, 2022 2.95 [2.12; 3.78] 7.8%
Random effects model 3.06 [2.55; 3.58] 21.9%
Heterogeneity: I^2 = 0%, τ^2 = 0, p = 0.92
Viral vector-mRNA
R.L. Atmar, 2022 2.61 [1.49; 3.73] 5.7%
R.L. Atmar, 2022 2.52 [1.48; 3.56] 6.2%
Random effects model 2.56 [1.80; 3.32] 11.9%
Heterogeneity: I^2 = 0%, τ^2 = 0, p = 0.91
Random effects model 2.96 [2.63; 3.30] 100.0%
Heterogeneity: I^2 = 77%, τ^2 = 0.1958, p < 0.01
0 1 2 3 4 5

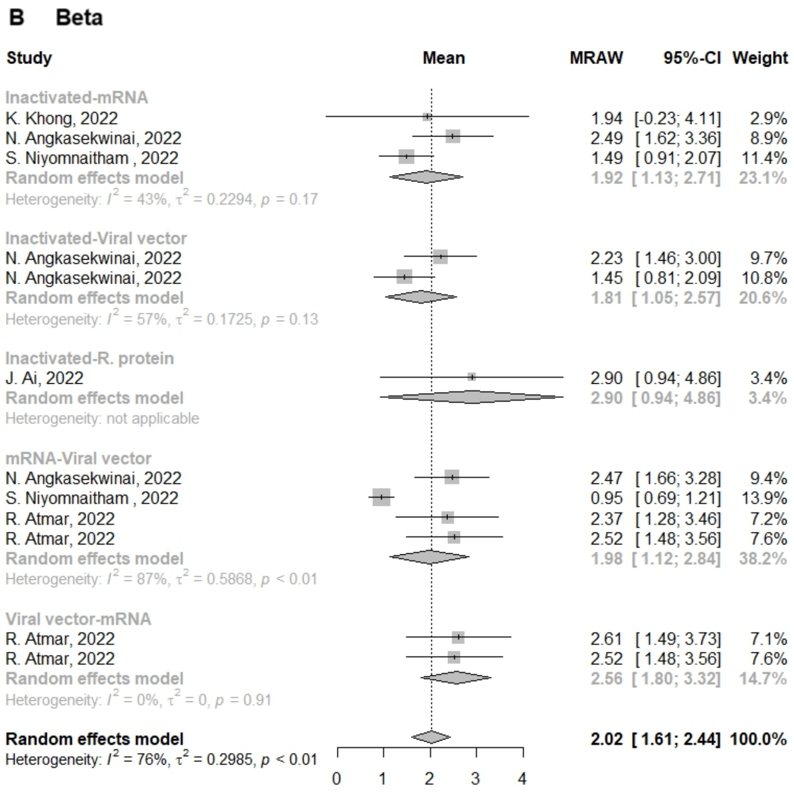
B Beta
Study
Mean
MRAW
95%-CI
Weight
Inactivated-mRNA
K. Khong, 2022 1.94 [-0.23; 4.11] 2.9%
N. Angkasekwinai, 2022 2.49 [1.62; 3.36] 8.9%
S. Niyomnaitham , 2022 1.49 [0.91; 2.07] 11.4%
Random effects model 1.92 [1.13; 2.71] 23.1%
Heterogeneity: I^2 = 43%, τ^2 = 0.2294, p = 0.17
Inactivated-Viral vector
N. Angkasekwinai, 2022 2.23 [1.46; 3.00] 9.7%
N. Angkasekwinai, 2022 1.45 [0.81; 2.09] 10.8%
Random effects model 1.81 [1.05; 2.57] 20.6%
Heterogeneity: I^2 = 57%, τ^2 = 0.1725, p = 0.13
Inactivated-R. protein
J. Ai, 2022 2.90 [0.94; 4.86] 3.4%
Random effects model 2.90 [0.94; 4.86] 3.4%
Heterogeneity: not applicable
mRNA-Viral vector
N. Angkasekwinai, 2022 2.47 [1.66; 3.28] 9.4%
S. Niyomnaitham , 2022 0.95 [0.69; 1.21] 13.9%
R. Atmar, 2022 2.37 [1.28; 3.46] 7.2%
R. Atmar, 2022 2.52 [1.48; 3.56] 7.6%
Random effects model 1.98 [1.12; 2.84] 38.2%
Heterogeneity: I^2 = 87%, τ^2 = 0.5868, p < 0.01
Viral vector-mRNA
R. Atmar, 2022 2.61 [1.49; 3.73] 7.1%
R. Atmar, 2022 2.52 [1.48; 3.56] 7.6%
Random effects model 2.56 [1.80; 3.32] 14.7%
Heterogeneity: I^2 = 0%, τ^2 = 0, p = 0.91
Random effects model 2.02 [1.61; 2.44] 100.0%
Heterogeneity: I^2 = 76%, τ^2 = 0.2985, p < 0.01
0 1 2 3 4

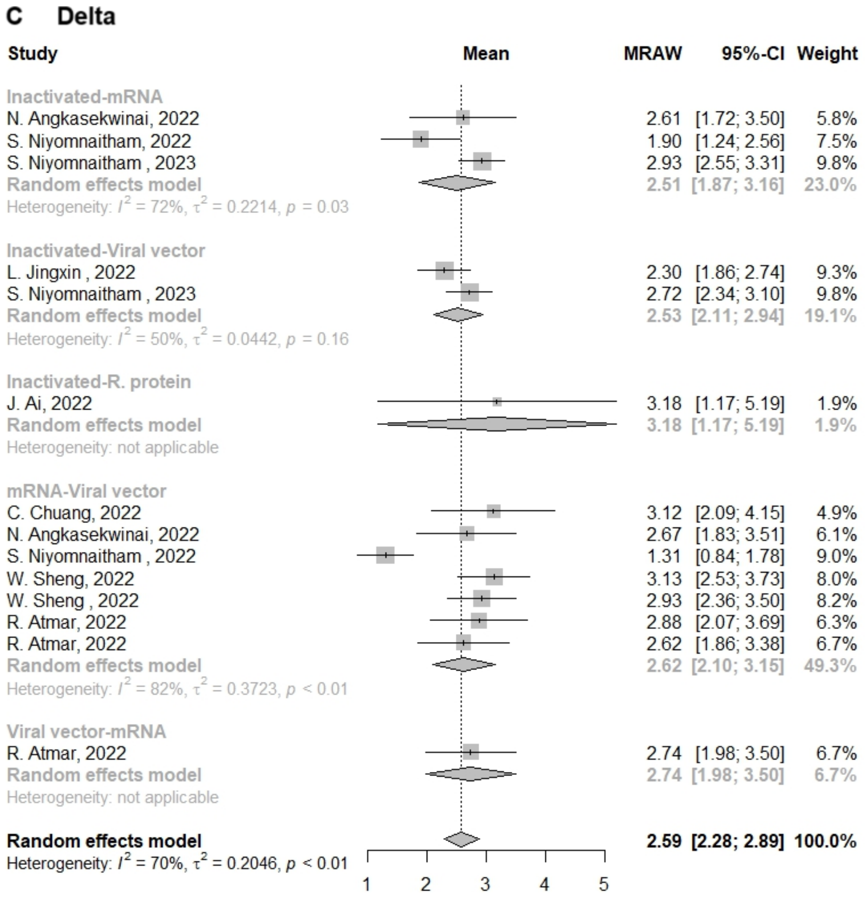
C Delta
Study
Mean
MRAW
95%-CI
Weight
Inactivated-mRNA
N. Angkasekwinai, 2022 2.61 [1.72; 3.50] 5.8%
S. Niyomnaitham, 2022 1.90 [1.24; 2.56] 7.5%
S. Niyomnaitham , 2023 2.93 [2.55; 3.31] 9.8%
Random effects model 2.51 [1.87; 3.16] 23.0%
Heterogeneity: I^2 = 72%, τ^2 = 0.2214, p = 0.03
Inactivated-Viral vector
L. Jingxin , 2022 2.30 [1.86; 2.74] 9.3%
S. Niyomnaitham , 2023 2.72 [2.34; 3.10] 9.8%
Random effects model 2.53 [2.11; 2.94] 19.1%
Heterogeneity: I^2 = 50%, τ^2 = 0.0442, p = 0.16
Inactivated-R. protein
J. Ai, 2022 3.18 [1.17; 5.19] 1.9%
Random effects model 3.18 [1.17; 5.19] 1.9%
Heterogeneity: not applicable
mRNA-Viral vector
C. Chuang, 2022 3.12 [2.09; 4.15] 4.9%
N. Angkasekwinai, 2022 2.67 [1.83; 3.51] 6.1%
S. Niyomnaitham , 2022 1.31 [0.84; 1.78] 9.0%
W. Sheng, 2022 3.13 [2.53; 3.73] 8.0%
W. Sheng , 2022 2.93 [2.36; 3.50] 8.2%
R. Atmar, 2022 2.88 [2.07; 3.69] 6.3%
R. Atmar, 2022 2.62 [1.86; 3.38] 6.7%
Random effects model 2.62 [2.10; 3.15] 49.3%
Heterogeneity: I^2 = 82%, τ^2 = 0.3723, p < 0.01
Viral vector-mRNA
R. Atmar, 2022 2.74 [1.98; 3.50] 6.7%
Random effects model 2.74 [1.98; 3.50] 6.7%
Heterogeneity: not applicable
Random effects model 2.59 [2.28; 2.89] 100.0%
Heterogeneity: I^2 = 70%, τ^2 = 0.2046, p < 0.01
1 2 3 4 5

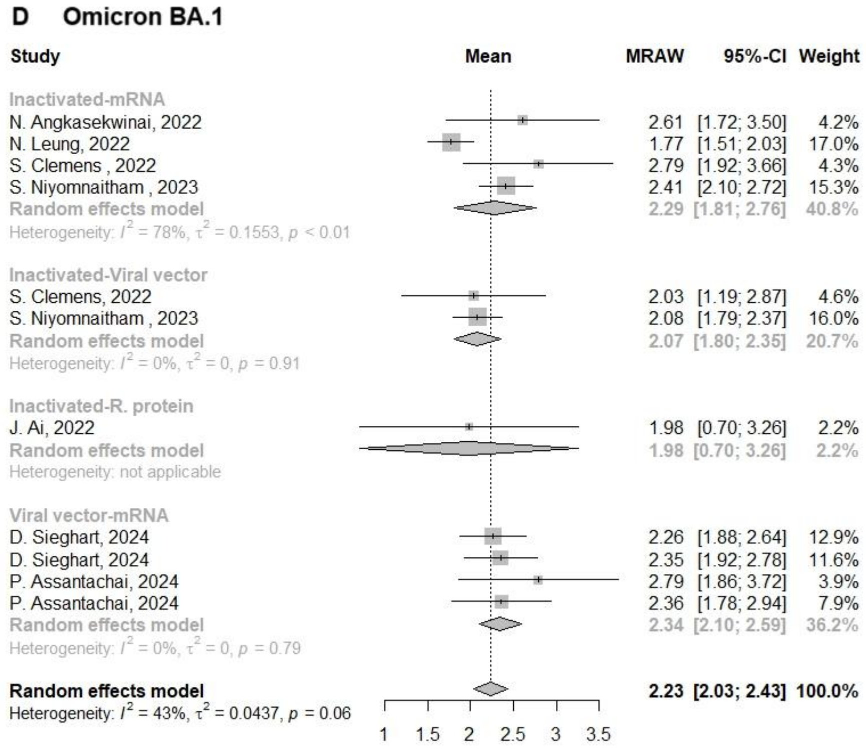
D Omicron BA.1
Study
Mean
MRAW
95%-CI
Weight
Inactivated-mRNA
N. Angkasekwinai, 2022 2.61 [1.72; 3.50] 4.2%
N. Leung, 2022 1.77 [1.51; 2.03] 17.0%
S. Clemens , 2022 2.79 [1.92; 3.66] 4.3%
S. Niyomnaitham , 2023 2.41 [2.10; 2.72] 15.3%
Random effects model 2.29 [1.81; 2.76] 40.8%
Heterogeneity: I^2 = 78%, τ^2 = 0.1553, p < 0.01
Inactivated-Viral vector
S. Clemens, 2022 2.03 [1.19; 2.87] 4.6%
S. Niyomnaitham , 2023 2.08 [1.79; 2.37] 16.0%
Random effects model 2.07 [1.80; 2.35] 20.7%
Heterogeneity: I^2 = 0%, τ^2 = 0, p = 0.91
Inactivated-R. protein
J. Ai, 2022 1.98 [0.70; 3.26] 2.2%
Random effects model 1.98 [0.70; 3.26] 2.2%
Heterogeneity: not applicable
Viral vector-mRNA
D. Sieghart, 2024 2.26 [1.88; 2.64] 12.9%
D. Sieghart, 2024 2.35 [1.92; 2.78] 11.6%
P. Assantachai, 2024 2.79 [1.86; 3.72] 3.9%
P. Assantachai, 2024 2.36 [1.78; 2.94] 7.9%
Random effects model 2.34 [2.10; 2.59] 36.2%
Heterogeneity: I^2 = 0%, τ^2 = 0, p = 0.79
Random effects model 2.23 [2.03; 2.43] 100.0%
Heterogeneity: I^2 = 43%, τ^2 = 0.0437, p = 0.06
1 1.5 2 2.5 3 3.5

Fig. S2. **Forest plot of the pooled log-transformed neutralization titers against the wild type (A) SARS-CoV-2 wild type, (B) Beta, (C)Delta, and (D) Omicron BA**. 1 after heterologous booster vaccination. MRAW, mean raw; 95%-CI, 95% confidence interval; I^2, index for the degree of heterogeneity between studies. τ^2, measure of heterogeneity. Weight is an indicator of the impact of each study on the overall results. Squares represent effect sizes for a single study, and rhombus represent pooled results for all studies.
